# The health and educational impact of removing financial constraints for school sport

**DOI:** 10.1177/1356336X221104909

**Published:** 2022-06-14

**Authors:** Lauren Denise Sulz, Doug Lee Gleddie, Cassidy Kinsella, M. Louise Humbert

**Affiliations:** 3158 University of Alberta, Canada; 3158 University of Alberta, Canada; 3158 University of Alberta, Canada; 7235 University of Saskatchewan, Canada

**Keywords:** School sport, comprehensive school health, youth, low-income families, socioeconomic status

## Abstract

Financial barriers often restrict sport participation among children from low-income families. Schools are thought to offer equitable access to programming, including school sport participation. However, pay-to-play school sport models can inhibit participation among students from low-income households. Recognizing the potential benefits of school sport and realizing the financial barriers to participation, the purpose of this study was to understand the extent to which school sport promotes educational experiences and holistic well-being of Canadian youth from low-income families. A case study was conducted with stakeholders who were supported by funding from a non-profit organization to help cover the costs of school sport registration fees. Data were collected from in-depth interviews with low-income students and their parents, teacher-coaches and school administrators. Three overarching themes were representative of the experiences of school sport participation among low-income students: (1) healthy student-athletes, (2) developing student-athletes in school, for life, and (3) supporting student-athletes as a community. The participants perceived that school sport participation offered holistic health benefits, and developed skills and behaviours that support positive educational experiences and foster life skills. Further, our results highlighted the importance of the school community in supporting low-income students to participate in school sport teams and the need to reframe school sport to better support low-income families.

## Introduction

Youth participation in sport has been associated with educational and health benefits (Bailey et al., 2009; Brière et al., 2020; Moeijes et al., 2019). Youth who participate in quality sport programmes often experience higher levels of school engagement and connectedness, improved emotional regulation, higher levels of physical activity participation and develop important life skills (e.g. communication, goal-setting and teamwork) (Eime et al., 2013; Holt et al., 2008; Neely and Holt, 2014; Yanik, 2018). However, children from low-income families are less likely to participate in sport due to financial barriers (Holt et al., 2011; Tandon et al., 2021). The school is a setting that purports to offer convenient and equitable access to sport, yet pay-to-play school sport (SS) models inhibit participation among students from low-income households (Holt et al., 2011; Somerset and Hoare, 2018) due to a lack of financial resources available within a family to support participation in extracurricular activities, such as SS (Snellman et al., 2015). In pay-to-play SS models, parents are required to pay registration fees for their child(ren) to participate in SS programmes (Eyler et al., 2018; Holt et al., 2011). Pay-to-play fees are one action taken towards assisting schools to fund the cost of SS (e.g. uniforms and travel) and adapt to budget shortfalls within the education system (Clark et al., 2012; Zdroik and Veliz, 2016). In Canada, for example, a cost is associated with SS to cover out of town travel, team fees for competition, team meals and apparel (Holt et al., 2011). Similarly, in the United States, 60% of schools have fees associated with SS (Clark et al., 2012). These pay-to-play models are concerning for low-income families, as the reduction of extracurricular opportunities affects students’ access to peer relationships, exposure to adult role models and connection to their school community (Snellman et al., 2015). School connectedness, facilitated by SS, is positively associated with school motivation (Gonida et al., 2009), social and emotional functioning (Brière et al., 2020) and academic achievement (Gillen-O’Neel and Fuligni, 2013) and negatively associated with school dropout rates (Hascher and Hagenauer, 2010).

Additionally, socioeconomic status (SES) is one of the most significant social determinants of health (Quon and McGrath, 2014). Student health is critical as there is a relationship between health and academic achievement, where healthy students are better learners (Basch, 2011). Unfortunately, youth from low-SES families have poorer access to, opportunities for and availability of physical activity (Hankonen et al., 2017; Humbert et al., 2006). This is problematic as physical activity is associated with better mental well-being or reduced symptoms of mental health disorders in youth (Bell et al., 2019). Other short- and long-term health benefits include psychological outcomes, such as higher self-esteem, resilience, an enhanced sense of belonging and lower rates of depression (Bailey et al., 2009; Brière et al., 2020). Jewett et al. (2014) examined the association between participation in SS during adolescence and mental health in early adulthood. Findings showed SS as a statistically significant predictor of lower depression symptoms, lower perceived stress and high self-reported mental health in young adulthood. In addition, Holt et al. (2008) and others (Camiré et al., 2009; Trottier and Robitaille, 2014) found that life skills, interpersonal and intrapersonal skills that enable individuals to manage demands, can be learned through quality SS engagement.

Recognizing the potential benefits of SS and realizing the barriers to participation for low-income families, our research aimed to understand the experiences of participation on SS teams among students from low-income households. The specific objectives were to (a) explore the perceptions and experiences of low-income families involved in SS and (b) understand the perceived impact of participation on students’ holistic well-being (physical, social, emotional and mental) and educational experiences (e.g. connection to school and peers, attitudes towards school and perceived academic impact). We define educational experience as any interaction, learning and/or participation in curricular or extracurricular programming and/or other experience that takes place within the school community. A more comprehensive understanding of the role of SS in educational experiences and the well-being of low-income students provides opportunities to advance both sport and educational practice.

## Context

The study was conducted in a Western Canadian province where SS is most often coached by teachers with no associated compensation or contractual obligation. Rather, in many Canadian schools, there is a ‘moral obligation’ to volunteer in order to sustain SS programmes (Camiré, 2015). Canada's mission and vision for SS is to enhance the educational experience of youth (School Sport Canada, 2013). However, this vision and mission is not consistent across practice, as competition and winning can surpass student-athlete development, positive educational experience and well-being (Sulz et al., 2021). Unfortunately, recent empirical data on the cost of SS in Canada is limited. Within Canadian high schools, Holt et al. (2011) reported SS fees range from $400 to $450 per season/student for one major sport (football, basketball and volleyball) and $150 to $200 for sports such as handball and soccer. Although not explicitly referring to SS, the Canadian Youth Sport Report (2014) indicated parents/guardians were spending nearly $1000 annually per child on sport, highlighting the cost of youth sport for Canadian families. Similarly, families in the United States spend, on average, $693 annually per child for one sport (Aspen Institute, 2019). Although there are some funds available to help youth overcome financial difficulties in sport participation they are trifling.

## Frameworks

In order to explore the experiences of low-income students participating in SS from an educational and holistic health lens, we utilized two theoretical frameworks to guide our work: ecological systems theory (Bronfenbrenner, 2009) and the Comprehensive School Health (CSH) framework (Joint Consortium for School Health [JSCH], 2012). Ecological systems theory allowed us to explore the multiple levels of participants’ experience with SS and CSH enabled an understanding of the role of sport within a healthy school community.

### Ecological systems theory

Demonstrating that there are distinct yet interrelated systems that affect human behaviour, ecological systems theory allows exploration of simultaneous effects of individual traits, interpersonal and contextual factors (Bronfenbrenner, 2009). Ecological approaches have been successfully used in other youth sport studies (Holt et al., 2008, 2011) using the theory that individuals interact with multiple levels of human ecological systems. The microsystem is the most proximal human ecological system and comprises participants, a physical domain, a location and/or a programme of activities. The mesosystem encompasses different interactions/relationships between two or more microsystems (e.g. relationship between coach and child). The exosystem involves links between the social settings that do not directly involve the child but still affect them (e.g. parental job loss). The macrosystem is the most distal system and is composed of cultural and societal forces such as public policy, governments and economic systems (Bronfenbrenner, 2009).

### Comprehensive school health

CSH is an internationally recognized framework for supporting improvements in students’ health and educational outcomes (JSCH, 2012). A CSH approach emerged in response to the recognition and understanding of the importance of multifaceted approaches to health and the association between health and learning (Kolbe, 2019). Researchers suggest moving from practices that rely mainly on a singular approach (e.g. health class and school policy) to a multi-pronged whole-school approach in order to improve the current health of youth (JSCH, 2012). The CSH approach integrates multiple school components that can improve both health and education outcomes and using this lens enables an understanding of the place of sport within a healthy school community.

## Methodology

### Study design

KidSport is a national non-profit organization in Canada that provides financial assistance to help cover sport registration fees for young people who live at/below the poverty line. Within the local region, KidSport subsidizes fees associated with participation in SS for approximately 260 students per year. Although there have been papers published on the impact of support systems for low-SES youth, most have focused on support for community or club sport (e.g. Holt et al. 2011) and not SS.

Case study methods require a defined ‘case’ (Yin, 2009); for this study, the case is defined as those stakeholders who are impacted by KidSport funding for SS participation within a local region in Western Canada: students, parents, teachers and administrators. Case studies also allow for the inclusion of a wide variety of information as data (Yin, 2009). In this study, semi-structured focus groups and individual interviews were used as the source of data collection engaging multiple stakeholders within the case. This process also allowed for alignment and analysis through both the ecological systems theory and CSH frames.

### Participants

Purposeful sampling (Patton, 2002) was used to identify families that had received KidSport funding, and these families were then asked to participate in this study. To be eligible for KidSport funding, applicants must be living within a low-socioeconomic bracket, based on Statistics Canada Low-Income Cut-Off + 30%, and participating on an SS team. In Canada, the low-income cut-offs after tax are income thresholds below which a family will likely devote a larger share of its income to the necessities of food, shelter and clothing than the average family. For a four-person Canadian household, for example, the low-income cut-off after tax is $41,406 (CAD) in urban communities (population 500,000 and over) (Statistics Canada, 2021).

Participants in this study (*N*  =  32) were all recruited through KidSport and included students (*n* = 12), parents (*n*  = 12), teachers (*n*  =  5), administrators (*n*  =  2) and a success coach^1^ (*n*  =  1). Student and parent participants were recruited via an email sent to all parents receiving a KidSport subsidy (approximately 100 families). Student participants played on a variety of SS teams, including soccer, volleyball, rugby, cheer, football, basketball and badminton. Some students participated in more than one SS team. Student participants were in grade 7 (ages 12–13) (*n*  =  4), grade 8 (ages 13–14) (*n*  =  3), grade 9 (ages 14–15) (*n*  =  1), grade 10 (ages 15–16) (*n* =  3) and grade 12 (ages 17–18) (*n*  =  1) at the time of the study. Once we identified the participant families, KidSport contacted the appropriate teachers, administrators and the success coach at their schools to participate in the study.

### Data collection

Prior to the commencement of the study, ethical approval was obtained from the university and school boards. All participants completed consent and assent forms. Semi-structured focus groups and individual interviews were used as the source of data collection. Three focus groups were conducted with student participants (3–5 students per group) and three focus groups were conducted with parent participants (3–5 parents per group). Data were collected from teachers through two focus groups (2–3 teachers per group), from administrators through two individual interviews and from the success coach through one individual interview. All focus groups lasted approximately 45 to 60 minutes and individual interviews lasted approximately 30 to 45 minutes.

A semi-structured interview guide was developed for each participant group: (a) students, (b) parents and (c) school stakeholders. The interview guides were informed by ecological systems theory (Bronfenbrenner, 2009) with questions including levels of influence (e.g. how has playing on an SS team impacted the relationships in your life?) and the CSH framework (JSCH, 2012) with questions related to education and health (e.g. has playing on an SS team impacted the educational experiences of your child?). To avoid confusion with club or community sport, SS was first defined by the interviewer as ‘school-sponsored sport practiced outside regular class hours in which students participate in organized interscholastic games and competitions’ (Camiré and Kendellen, 2016).

### Data analysis

Focus groups and interviews were audio recorded, transcribed verbatim and participants were anonymized. Reflexive thematic analysis was used to identify core meanings and themes and helped to develop a narrative explanation that could account for and accurately describe the phenomena (Braun and Clarke, 2019). The process allowed the coding/theming to remain iterative while also drawing on the general structure provided by ecological systems theory and CSH. Initial codes were created based on the patterns and meanings that stood out in the data set and then organized into candidate themes. In the initial coding phase, segments of the data which appeared of interest to the analyst were highlighted, and then codes were applied. After relevant data items were coded, the researchers actively interpreted the relationships among the different codes and examined how these relationships may inform the description of a given theme. That step resulted in 14 initial codes; however, there was a degree of overlap in the initial codes (e.g. ‘sense of purpose’ and ‘sense of belonging’) and codes were grouped together in clusters of related codes. The researcher team reviewed and deliberated to assemble codes into four cohesive candidate themes (‘Healthy Students’, ‘Lifelong Skills’, ‘School Skills’ and ‘Community Support’). Once the candidate themes were devised, the themes were reviewed and refined to finalize the overarching themes to represent the patterns in the data set. The candidate themes ‘Lifelong Skills’ and ‘School Skills’ were closely related, with one identifying transferable skills learnt from SS to life and the other discussing skills that transfer to school. This resulted in the creation of one main theme representing skills learned from SS, with two distinct subthemes, ‘Life Skills’ and ‘School Skills’. Sufficient evidence was gathered from the data set and organized under each theme, as well as for their subthemes. Finally, themes were described in order to synthesize what each theme was about and to describe what part of the data that each theme represented (Braun and Clarke, 2019).

## Results

Three overarching themes were representative of the experiences of SS participation among low-income students: (1) healthy student-athletes, (2) developing student-athletes in school, for life, and (3) supporting student-athletes as a community.

### Theme 1: Healthy student-athletes

Participants perceived that multiple aspects of health can be developed through SS experiences. They understood that students who engaged in SS programmes received holistic health benefits, including benefits associated with physical, emotional, social and mental health:

We obviously know the holistic impact of sport being mental, emotional, and obviously physical … but the mental and emotional [impact] that's a part of the developmental needs of teenagers. The idea is that [school sport] is going to build camaraderie, what it means to respect somebody on the court or how to respect your own body or how to be healthy or how to engage in cooperation. (Sharlene, Teacher-Coach)

Students recognized the benefit of participating on SS teams to their overall level of activity. One student stated:

It benefits me because I know I wouldn’t be as active, if I didn’t participate in SS. (Zoya, Student)

Similarly, parents mentioned that SS participation positively impacted their child's physical activity behaviours and their child's desire to be engaged in physical activity in their free time:

I think the big one for me is the activity level and them wanting to be active. (Isabella, Parent)

Throughout the interviews, participants discussed how the health benefits of SS participation extended beyond the physical to include emotional and mental health. For example, parents had a particular interest in making sure their children had a positive way to release emotions, and perceived that SS provided them with an avenue to do so. One parent described her daughter’s experiences using physical activity as an emotional outlet:

I think my daughter wouldn’t have an outlet, like she dances when she's happy, when she's mad or she's feeling energetic, so I would say I think she probably wouldn’t have a positive outlet, a healthy outlet for her emotions. (Carla, Parent)

Further, school stakeholders noted that SS experiences offer students opportunities to experience a variety of emotions, positive and negative, such as winning and losing, failure and success, which allows for, the development of positive emotional and mental health. When reflecting on this, adult participants discussed the outcomes developed through SS participation that transferred to other contexts and situations:

I think emotionally, in a training session or a game, a player is going through so many different highs and lows. And it helps them to self-regulate themselves, and it allows them to work on that mental perseverance. And I think it can transfer whatever happens on the field; it can also transfer through life, like if you’re going through whatever adversity, it can help them. (Luis, Teacher-Coach)

Participants also discussed the impactful role of SS on students’ social health. Students living in low-income households are often left alone afterschool due to the financial and employment demands of their parents. It was noted by school stakeholders that SS provides a second family for these students and a safe place to be during after school hours:

…maybe an unknown thing about SS is that many of our kids are going home to empty houses. Mom and dad aren’t around or might be even disengaged or mom is working two jobs ’cause there is no dad or vice versa. And that's a pretty lonely place to go to after school, it's pretty isolating, it's pretty disheartening, that's pretty sad. But now five days a week, practicing for a couple months with this family of people gives you a life to have. You feel a part of something, you have a responsibility, you have an accountability, it's my job to show up, the team needs me. (Samuel*,* Success Coach)

Being a part of a team, there's a unique dynamic to that. The fact that you’re wearing the same jersey, you’re wearing that same logo, you feel a part of a family. And specifically, here in this building (school with a high population of low-income students), a lot of these kids might not have what we traditionally think of as a family, a good family. So, for them, we give them that stability, we give them that sense of purpose and family through sport and that is a really big advantage*.* (Jacqueline, Teacher-Coach)

Participation also helped students interact and form meaningful relationships with other students. The social circle that students are embedded in when they are involved in SS fosters social connection both during and outside of sport; without SS, this connection would be greatly diminished. This is highlighted by the following quotes:

I think since we all go to the same school and we get to see each other more often that our bonds are stronger. I think we’re just stronger together because we get to see each other every day. (Rowan, Student)

The group of friends he has, he's had them since we moved here five years ago, so there's a group of about six of them that went to junior high together and now in high school together they were on the junior football team last year and the basketball team and now they're on the senior football team this year … he wouldn't have those [friends], like guarantee, he wouldn't be in that same circle of friends and have that same closeness if it wasn't for football. (Max, Parent)

School stakeholders also discussed the role sport has in integrating students into the school community and developing a sense of social belonging. They perceived that SS bridges student economic differences and develops shared experiences to enhance social well-being:

Well, the cool thing about that is, when you put on that jersey doesn’t matter where you were born, it doesn’t matter where you’re from. The only thing different is the number on your back but everyone's the same when they’re on the field. (Ken, Teacher-Coach)

I always think it must be so interesting but I’ll see it, at our big pep rallies they’ll introduce the football team, I would see our more popular and well-adjusted and top athletes and all that and that's great but then to see a kid from a less privileged family go and join them and stand beside them wearing the same jersey, like in that moment they’re teammates and it doesn’t matter what class he has, it doesn’t matter who's at home. They’re the same, on the same team, with the same goal. (Sharlene, Teacher-Coach)

Participants also discussed that SS participation kept students from getting involved in other activities that may have been detrimental to their health and well-being:

I think it gives us all something to do instead of doing something that's like bad, you go and do something that's fun and benefits us in many ways. (Carlos, Student)

I think having a place for kids to go after school is a positive thing, and you know you look at two kids that come in as grade 10’s first day of school, they have similar backgrounds, similar junior highs, one plays football for three years, and the other goes to the mall for three years. Is one better than the other? I think so. (Luis, Teacher-Coach)

### Theme 2: Developing student-athletes in school, for life

The second theme was centred around the impact of SS on student development. Specifically, two subthemes were generated from the data: (a) development of school skills and (b) development of life skills.

#### Development of school skills

This sub theme is related to the impact of SS on academic behaviours. A number of participants discussed how students’ attitudes towards school were improved due to their involvement in SS. Student participants explained that SS enhanced their desire to attend school and succeed academically:

It makes me really want to succeed, you would want to go to class way more and do better. (Sophia, Student)

…whenever I do SS, it does make me have a bigger drive to do homework more and do better in classes ’cause I know that right after school I have to go and hurry up and go to my sport and if I didn’t have that, I’d be lazy to my homework or be slow doing it in class. (Luca, Student)

Parents also noted their child's interest in going to school was increased due to their excitement and enjoyment of SS. A parent explained:

…he can’t wait to get to school and a lot of the time it's the sports. (Nicole, Parent)

School stakeholders also discussed that SS had positively impacted students’ attitude towards school and their experiences in school. They highlighted the importance of ‘getting them in the door’ through sport but keeping them at school for academics. A teacher-coach reflected on this:

It's great, they love coming to school for soccer. But the most important part is they’re coming to school for a sport, but they have to be here in the building. (Ken, Teacher-Coach)

An administrator stated:

The second thing is school engagement. Kids who are on school teams come to school right. That's just a fact. So if it's a way of keeping kids in the building here consistently and if we know that it's a tried and true way, then we’re absolutely going to use that. (Johanna, Administrator)

It was clear that school stakeholders viewed SS as a way to keep students accountable for their attendance and academics. SS was viewed as a privilege that can be removed if students were not attending and/or doing well in school. According to the school stakeholders, the SS programmes were designed for students to succeed in both athletics and academics:

Part of my role is to keep track of the students’ academic standing, so whether they’re showing up to school, whether they’re on time, if they’re late, how they’re doing in their classes … and if they’re missing assignments. I’m kind of hounding them, on top of them … in our SS programme we can have that carrot, that token at the end of the day to say to them; okay well you’re missing an assignment here, you need to make sure that's handed in or else you’re not coming with us to the sport facility. Now what ends up happening is academic success because they start to just realize that let's just start getting the habit of doing it properly. (Oscar, Administrator)

For parents, an added benefit of SS was having a teacher in their child's school invested in their child's academic behaviours. Parents felt comfortable trusting the coaches to support their children and felt that when they were also their teacher, they were receiving extra support to do well academically. Parents voiced that the teacher-coaches were invested in their child's academic success:

…by having the teachers know what's going on as coaches and in the classroom and in the hallways, it's even better because they are building the character of those children. (Max, Parent)

…especially with my daughter on the soccer team at school, her marks have to be there so that also the accountability to her coaches to be checking in on where she's at academically as well. (Dillion, Parent)

Students also knew that due to their relationship with their coaches they could reach out to them if they were struggling in school and felt like they could trust them to give them the help they needed. Teacher-coaches provided a supportive relationship that some students reported was more like having a parent or guardian at school than a teacher:

I know that they will always be there for me and if I ever need them, they will always guide me for something, like if I ever need help sports-wise, just life-wise I know I can go back to them and ask them for anything; they always have something good to say. (Gianna, Student)

…my daughter and the coaches, there's a bond, like if they’re struggling with something and it doesn’t necessarily have to be sports or school, they feel confident going to the coach and saying hey you know what I’m struggling right now. (Kirsty, Parent)

#### Development of life skills

Students perceived that experiences with SS helped them to develop life skills that they can transfer into other contexts. School stakeholders expressed their understanding that students from low-income families were provided opportunities to develop important life skills through their experiences on SS teams:

I think every single kid that's a part of our athletic programme is learning lessons about what it means to be a good teammate; again that accountability piece, communication piece, I think those are premiums that we put on all of our programmes, and there are so many positive things involved with being a part of a sports team. (Sage, Teacher-Coach)

Parents in particular made specific mention of the increased confidence their children displayed and attributed this to SS participation:

I see my daughter being more assertive, more confident. (Johanna, Parent)

The students also recognized that their experiences with SS enhanced their confidence both within their sport and in life:

Sports builds up confidence a lot. Coming to grade seven I didn’t have the kind of confidence I have right now, and throughout those three years I’ve become a better player because of my confidence and I feel like sports helps you build confidence and confidence is pretty much everything, if you have the confidence to do something, you’ll do it. (Omar, Student)

Other life skills discussed by participants included self-discipline and goal-setting. For example, one student stated:

Discipline just came with it [playing SS] ’cause the coach would hold you up to a standard, so it just helps you in real life instead of just in your sports life. (Zoya, Student)

Parents identified how their children were goal oriented because of their involvement in SS and sport helped their children understand that in order to achieve success they needed to be self-disciplined and goal oriented. This was evident in the following parent comment:

He's got a goal, he's driven, there's motivation, there's ambition, there's a path, like we’re trying to achieve something. (Sherry, Parent)

Communication and developing relationships were also noted as skills that were further developed through SS. One student stated:

It makes me more social because I have to communicate with my team and put my thoughts out there. (Karina, Student)

Students also described their difficulty building relationships with their teachers and establishing a sense of trust with adults. It was apparent that through their participation on SS teams, students developed new and more positive relationships with their teacher-coaches. These relationship skills also allowed them to open up to their coaches and engage in conversations about their lives outside of sport. Two students explained:

When you’re going through hard times, you can tell the coach what you’re going through and he can help you out. Like kind of like trust issues, basically. You know that you can trust him. (Gianna, Student)

And like they’re always open to everything, so you can talk to them about anything, not just soccer, but like anything else that you’re having problems with. (Luca, Student)

### Theme 3: Supporting student-athletes as a community

We found that although community support is needed for low-income students to participate in SS, their participation also brings the school community together. The cost of SS was a substantive barrier to participation and without support from the school community, families would not be able to participate. Financial barriers to participation were discussed by all participant groups, including students. One student described their worry about the financial burden, the cost of SS places on their family:

I worry about it, even though I am not supposed to be worrying. (Karina, Student)

Another student echoed these sentiments in the following quote:

I have five sisters and one older brother, so there's a lot of funds to be paid. (Carlos, Student)

One central idea was that schools and teacher-coaches just ‘make it happen’ with regard to overcoming financial barriers to participation among low-income students. An administrator stated:

We will not have costs be the reason why a student doesn’t access SS. (Johanna, Administrator)

It was also noted within the interviews and focus groups that parents have the best intentions to pay but at times cannot come up with the money. In these situations, the schools end up paying the SS fees. One school administrator explained:

He says he has a plan and then maybe it falls through. I hear ‘My dad's going to get the child tax and we’ll have the money and okay okay…’, and then the season's over and it goes on their school fee sheet and then at the end of the year we say ‘you know you got to get those school fees taken care of before grad’. And at the end of the day, the school just eats it. (Oscar, Administrator)

In some cases, teacher-coaches would set up an agreement with other community organizations for the student to pay off the registration fees and other costs associated with SS participation. For example, a teacher-coach described how students at his school were able to work to help pay off SS fees:

I know we have a tremendous need in our building for kids who can’t afford to pay fees, but we’ve taken upon ourselves to do the little extra pieces to get kids working off fees within the school so they can play and you know we’ve made the time to do that, it does take time though right. (Sage, Teacher-Coach)

Additionally, it was discussed that teacher-coaches would promote organizations like KidSport to assist with SS registration fees. This was shown in an excerpt from a success coach:

I went down into the dressing room, and I explained the process and kind of the ways that KidSport could help them. And kids that identified themselves as ‘yeah that would be something that I need’ would then come and approach me. (Samuel, Success Coach)

Parents highlighted the need for organizations like KidSport to help with the cost of SS participation. Consistently discussed within the parent interviews was their understanding that their child would not be able to play SS without the financial support from KidSport. This was noted in the following quote:

I do find that KidSport really does help offset the fees ’cause I told her you wouldn’t be a cheerleader for the school because I mean the gear and the equipment and the shoes and the competition fees and like competition fees alone are 165 dollars. (Barb, Parent)

Although it takes a community to help overcome financial barriers to SS participation among low-income students, the participation in SS by low-SES students was described as contributing to the development of the school community. Teacher-coaches and administrators discussed the sense of community SS builds for low-income families:

Parents are connected to the school because their kids are connected, and they’re having to show up to games; they’re picking them up after practice, meeting the coaches. High schools typically are known across the board to have low parental involvement by this stage of the education game. Like a lot of things are done online, parents don’t have to come in and volunteer in the classroom anymore. But that keeps a foot in the door for these parents to continue to connect with us. So you take SS out, now you’ve got less parents in the school. (Jacqueline, Teacher-Coach)

Parents and students also echoed the increased sense of community and family through their involvement in SS. Students voiced an enhanced engagement in the school and involvement in other school activities. Parents felt they were part of a larger community of other sport families:

It also makes me or helps me participate more in my school in general because I’m already part of a school event – why not be a part of more? (Fabian, Student)

Community-wise, it just really made us feel like we’re a part of our northeast family because there's such a crossover. The parents, they’re watching the kids in the school setting as well as your friends and parents being at award ceremonies, like everyone cares about the kids. (Derek, Parent)

## Discussion

The purpose of this research was to understand the experiences of participation on SS teams among students from low-income families. Using the dual lenses of the ecological systems theory and the CSH framework enabled an exploration of multiple levels and an understanding of potential educational and health impacts of participation. Overall, we found that SS participation provided positive experiences for students from low-income families. Specifically, SS involvement positively influenced the perceived health and development of these students. Our research yields important information about the benefits, challenges and opportunities associated with SS among low-income families. A more comprehensive understanding of the role of SS for educational experiences and the well-being of low-income students provides opportunities to advance both sport and educational practice. Although students from low-income households are often less likely to participate in extracurricular activities, they often experience greater benefit when they do (Heath et al., 2018). While the benefits of youth involvement have been examined for the ‘general’ school population (Bailey et al., 2009; Holt et al., 2008), few studies have focused on specific benefits of SS for students from low-income families, in particular the impact of participation on educational experiences. Our findings highlight the potential benefits of SS participation among youth from low-income families and we recommend advocating for SS participation from both an educational and health perspective. Considering the positive impact of SS, our findings reinforce the important role of SS within a healthy school community.

Our findings illustrated physical benefits of SS participation are in line with earlier research that showed youth participation in sport positively impacts the physical well-being of participants (Janssen and LeBlanc, 2010). These results add to the limited literature on the physical health benefits of SS (Bocarro et al., 2008; Dudley et al., 2011) and uniquely contribute to sport literature by specifically exploring holistic health benefits to youth from low-income families. Emotional and social benefits from SS were also discussed by participant groups. These benefits included the development of meaningful relationships with teacher-coaches and teammates, increased emotional support and an enhanced sense of belonging and purpose. Low-income students often are home alone after school while parents and guardians are at work. As such, SS provides students with a second family, social and emotional support after school hours, and enhanced relationships with peers and teacher-coaches. Further, participants discussed the positive impact SS had on students’ mental well-being. A recent systematic review and meta-analysis (Andermo et al., 2020) examined effects of interventions targeting school-related physical activity on mental health in children and adolescents. Findings showed there was a significant beneficial effect of school-related physical activity, in this case SS, on resilience, positive mental health, well-being and anxiety. The authors concluded that findings may reinforce school-based initiatives to increase physical activity (Andermo et al., 2020). Our results support the benefit of SS programmes and highlight the need to view SS participation as benefiting the holistic well-being of youth beyond physical benefits.

With regard to the skill development of students through SS participation, our findings are consistent with life skills in sport research (Camiré et al., 2009, 2013; Holt et al., 2008). We found that students perceived that they developed life skills such as teamwork, commitment and responsibility through SS participation. Camiré et al. (2009) explored high school students’ beliefs of learned life skills through SS and the transfer of these skills to other life domains. The authors found that the students believed that they had the opportunity to develop a number of life skills through SS participation and these skills could be transferred to other life contexts. Unique to our study is the perceived contribution of SS participation to educational experiences and educational behaviours of students. The benefits to academic performance associated with involvement in community youth sport are documented within the literature (e.g. Logan et al., 2019) and Wretman (2017) demonstrated a direct positive association between SS participation and academic achievement. However, our research enhances our understanding of why students’ academic achievements may be impacted by SS participation, in particular among low-income youth who often display lower achievement levels and negative educational experiences (Ferguson et al., 2007; Reardon, 2013). Participants shared that SS provided connection to school – a sense of belonging and purpose – and motivation to attend school, which they perceived positively impacted their academics. Furthermore, teacher-coaches reported SS kept students at school preventing unwanted behaviours. When parents were asked about the impact of SS on their child's education, several noted the addition of a caring adult in the school, invested in their child's academic behaviours, an increased desire to do well, and that their children were going to school with excitement and a positive attitude. These findings emphasize the valuable contributions SS participation can have on the educational experiences of students from low-income families.

The development of educational skills and positive educational experiences, coupled with the reported holistic health benefits, emphasize how SS aligns with a CSH approach. Demonstrating how SS programmes can contribute to healthy school communities is a unique and important implication of this work. Most CSH initiatives and programmes do not include SS as a component of their whole-school approach aimed to improve health and education outcomes for children and youth through improvements to the school community (e.g. Beaudoin, 2011; Veugelers and Schwartz, 2010). For example, in a review of whole-school health approaches in high schools by Sulz and Gibbons (2016), including an analysis of targeted CSH components, none of the whole-school health approaches reviewed included SS as part of the CSH approach. Our findings indicate that SS can be a catalyst for health – especially important for low-income students as children from those families have increased health risks (Cohen et al., 2010; Gupta et al., 2007) and lower educational attainment and success (Ferguson et al., 2007). CSH approaches should consider SS as a viable element to reach the goal of improving health and education of school-aged children.

Within this study, parents indicated their child would not have been able to participate in SS without financial aid. This aligns with the literature on barriers to sport participation for students in low-income households (Holt et al., 2011; Somerset and Hoare, 2018). For example, Holt et al. (2011) examined low-income parents’ perceptions of the challenges associated with providing children sporting opportunities. Results showed that parents needed additional financial support in order to pay associated costs of sport participation. The authors noted that subsidization of youth sport programmes is limited due to the majority of funding being directed towards elite sport (Holt et al., 2011). Additionally, Tandon et al. (2021) reported that children from low-income families reported fewer days per week of physical activity, fewer sports sampled and lower rates of playing sport. The authors suggested that targeted solutions can support access for marginalized groups including the development of an intentional set of community-based strategies to eliminate barriers related to cost. It was evident from the focus groups that coaches and administrators were willing to support students whose families could not afford SS. In some cases, the school just quietly covered the cost. One teacher-coach indicated that students could ‘work off fees’. While this might disturb some, we see it as an indication of a willingness to support students who need it and a move towards schools as a setting in which sport is available to all students regardless of SES or other barriers (e.g. skill level). Schools can reach children in low-income areas and provide access to organized sport programmes. Sulz et al. (2021) suggested that SS should be re-imagined to better support all students and better align with the primary purpose of education systems – the development of contributing members of society. Given the context of public education, access should be universal, therefore SS should be accessible to all interested students. Moreover, our findings highlight the role SS can play in enhancing students’ feelings of inclusion, belonging and sense of family within a school community. SS provides an opportunity to help reduce socioeconomic inequities and enhance inclusion within the school context. As described by a teacher-coach, ‘all players wear the same jersey’. Redesigning SS for inclusivity will increase the number of low-income students who can play as well as enhance the quality of their sport experience within a welcoming and healthy school community.

## Conclusion

As we interviewed, read and reread the transcripts and engaged in thematic analysis we noticed one common thread, woven across all themes and subthemes – connectedness. Students who are ‘connected’ to their school community experience academic benefits, a reduction in hazardous behaviours and improved mental well-being (Blum, 2005; McNeely et al., 2002). Our findings in all three themes demonstrated that SS can play a key role in making students feel connected to their school community. The first theme, *healthy student-athletes*, revealed that students find their ‘sport family’ through involvement in sport at school. They make important connections and feel a sense of belonging that helps them to be healthier, happier and part of a team. The second theme, *developing student-athletes in school, for life*, also included subthemes of *school skills* and *life skills*. In both of these areas, having adult role models involved in SS provided both role models and another ‘advocate’ adult in students’ lives to help them build valuable skills and navigate obstacles. Finally, *supporting student-athletes as a community* speaks to not only the importance of building a community through sport programmes but also to the contributions those student-athletes make to the school community itself. In many cases, without the opportunity to get involved through SS, these children would not only be disconnected, but they would also be denied the chance to contribute their experiences and talents. Creating connection for students from low-socioeconomic backgrounds is critical, as young people from low-socioeconomic households experience greater adverse childhood experiences (Walsh et al., 2019). Therefore, for these students, connection to their school community through SS participation can be a key to mitigating detrimental outcomes to academic achievement and well-being.

### Pragmatic implications

All of the authors of this paper have been student-athletes, teacher-coaches/coaches and advocates for quality SS. As such, we do not want to neglect the pragmatic aspects of our findings and have some observations that may be helpful to those involved in SS. First, addressing the needs and barriers experienced by low-income students makes a difference – to their educational engagement and attainment, their health and their quality of life at school and maybe even at home. SS can have a particularly positive impact for these children who may lack a supportive home environment, other adult advocates in their lives and who struggle with poor health. KidSport is doing important work to support communities, families and children by helping pay for quality SS. Without support from outside organizations such as KidSport, many students from low-income families would simply be unable to afford the cost of entry. Our research has shown that the funding makes a difference. Supporting opportunities for SS helps low-income students succeed, not only in sport, but also in school and the community.

More generally, our findings lead us to recommend advocating for SS participation from an educational perspective – more specifically, CSH. According to our participants, SS not only improved the health of students but also enhanced their attitudes towards school, attendance and desire to achieve good grades. Considering SS as an integral part of a healthy school community, rather than an ‘extra’, could lead to policy changes such as reduced or no-cost play, more opportunities for *all* students to play (Sulz et al., 2021) and administrative backing of programmes. Furthermore, teacher-coaches are an essential element in the delivery and sustainability of SS. They act as another adult advocate for students, build community and support academic achievement. Ironically, teacher-coaches themselves are sorely lacking when it comes to support for the massive amounts of time and energy they give (Sulz et al., 2021). That needs to change.

Finally, the results of our research should be of interest to and have implications for local, provincial and national sport organizations that wish to better serve athletes from low-income families. Project findings allow policymakers to better understand economic obstacles to sport participation (school, community and ‘elite’) to reduce socioeconomic inequalities and be more inclusive. Rather than having students work off SS fees, or have the school quietly cover them, we can move to a system where sport is recognized as an integral part of a healthy school community – and funded as such. As teacher educators, we also call on teacher education programmes to recognize the benefits of SS and encourage teachers to get involved as part of their professional development and growth plans as educators. SS makes a difference – for school and for life.

The work to further our research on SS is not complete. We know that not all SS experiences are positive and our main goal is to help programmes get better – and serve more students. We want to continue to listen to those who are impacted by SS and learn from their experiences.

## Notes

1. Success coaches provide universal and targeted programming to the school community in order to promote mental health and well-being, enhance academic success and increase resiliency.
